# Cell fusion between gastric epithelial cells and mesenchymal stem cells results in epithelial-to-mesenchymal transition and malignant transformation

**DOI:** 10.1186/s12885-015-1027-1

**Published:** 2015-01-30

**Authors:** Xianghui He, Baosong Li, Yang Shao, Na Zhao, Yiling Hsu, Zhixiang Zhang, Liwei Zhu

**Affiliations:** 1 Department of General Surgery, Tianjin General Surgery Institute, Tianjin Medical University General Hospital, Tianjin, 300052 China; 2 Department of Anorectal Surgery, Affiliated Hospital of Binzhou Medical University, Shandong, 256603 China; 3 Department of Surgery, Feixian Hospital, Shandong, 256603 China

**Keywords:** Cell fusion, Carcinogenesis, Mesenchymal stem cells, Epithelial-to-mesenchymal transition

## Abstract

**Background:**

The discovery of cancer stem cells and tumor heterogeneity prompted the exploration of additional mechanisms aside from genetic mutations for carcinogenesis and cancer progression. The aim of the present study was to investigate the effect of cell fusion between mesenchymal stem cells and the gastric epithelial cells in tumorigenesis.

**Methods:**

Cell fusion between cord blood mesenchymal stem cells and human gastric epithelial cells was performed *in vitro*. Cell scratch and transwell assays were performed to determine migration and invasion abilities of the hybrids. The expressions of epithelial-mesenchymal transition-related proteins and genes were analyzed by immunocytochemistry and real time quantitative PCR. Tumorigenesis of the hybrids was evaluated through *in vivo* inoculation in nude mice.

**Results:**

Hybrids expressed the phenotypes of both donor cells. Aneuploidy was observed in 84.1% of cells. The hybrids showed increased proliferation, migration and invasion abilities compared with the parental cells. In addition, the expression of N-cadherin and vimentin in the hybrids was significantly higher than that of the epithelial cells, and the mRNA expression of the epithelial-mesenchymal transition-related genes, Twist and Slug, in the hybrids was also increased compared with that of the parental epithelial cells. Furthermore, the hybrids formed masses of epithelial origin with glandular structures in BALB/c nude mice.

**Conclusions:**

These findings suggest that cell fusion between gastric epithelial cells and mesenchymal stem cells may result in epithelial to mesenchymal transition and malignant transformation.

## Background

Carcinogenesis and cancer progression are multiple step processes that involve genetic mutation, cell-cell communication, and cell micro-environment interactions. Mutations are thought to be the principal pathway of malignant transformation [1]. Normal cells can accumulate mutations that are necessary for stepwise malignant transformation. However, the discovery of cancer stem cells and tumor heterogeneity widens the question regarding possible mechanisms of carcinogenesis and cancer progression. Cell fusion has been proposed as one of the possible mechanisms of carcinogenesis [2,3]. Mutations that are insufficient to transform on their own may combine through cell fusion to result in carcinogenic transformation. Some studies have shown that the expression of oncogenes or a mutated tumor suppressor p53 in one of the fusion partners is sufficient to produce heterogeneous progeny and result in oncogenic transformation [4].

Cell fusion between healthy differentiated cells is usually cytostatic and fails to generate oncogenic cells [5]. However, cell fusion produces a wide range of chromosomal aberrations, including chromosomal loss, chromosome disjunctions, and translocations [6]. The potential pathological consequence of fusion between bone marrow-derived stem cells (BMDSCs) and epithelial cells remains to be unknown. We previously hypothesized that fusion between an “altered” pre-malignant cell and a bone marrow-derived stem cell results in malignant transformation of the hybrid progeny cells [2]. The “altered” cells are defined as any cells with genetic or epigenetic changes sufficient to change the normal differentiation pathway of BMDSCs after fusion. In this study, we fused immortalized GES-1 cells with cord matrix-derived mesenchymal stem cells (CM-MSCs). GES-1 is a SV40-immortalized non-tumorigenic human gastric epithelial cell line [7]. GES-1 cells carry the SV40 T antigen and are non-tumorigenic when inoculated into nude mice. Mesenchymal stem cells (MSCs) are self-renewing stem cells residing in different tissues that can differentiate into multiple cell types, including osteocytes, chondrocytes, adipocytes, hepatocytes, myocytes, neurons and cardiomyocytes. The results revealed that the hybrids of GES-1 and CM-MSCs undergo epithelial-mesenchymal transition (EMT), indicated by the increased capability of proliferation, migration and invasion, and the expression of EMT-related genes and tumor formation in nude mice.

## Methods

### Ethics statement

Ethical and methodological aspects of the investigation protocols were approved by the Ethics Committee of Tianjin Medical University (Permit Number: TMUaMEC2008010).

### Cell lines

CM-MSCs were provided by Union Stem Cell & Gene Engineering Co. Ltd (Tianjin, China) and maintained in complete DMEM/F12 cell culture medium. GES-1, an immortalized and non-tumorigenic human gastric epithelial cell line established by Beijing Institute for Cancer Research, was generously provided by Dr. Chunsheng Kan from Tianjin Neurology Institute (Tianjin, China) and maintained in Dulbecco’s modified Eagle’s medium (DMEM) supplemented with 10% fetal bovine serum (Invitrogen, Beijing, China), 2 mM glutamine, 1 mM pyruvate, 50 μM 2-mercaptoethanol, penicillin (200 units/ml), and streptomycin (200 μg/ml) at 37°C in a 5% CO_2_ atmosphere.

### CM-MSCs and GES-1 cell fusion

Before fusion, GES-1 cells and CM-MSCs were cultured separately in complete DMEM/F-12 culture medium containing 10% fetal bovine serum (FBS). 5, 6- carboxyfluorescein diacetate succinimidyl ester (CFDA-SE, 2 μL/mL) was added to the culture medium of CM-MSCs for 20 min, following which the cells were washed twice with PBS and resuspended with culture medium. GES-1 cells were stained with PHK-26 according to the manufacturer’s protocol (Sigma-Aldrich, MO, USA). In brief, 2 × 10^7^ cells were collected and resuspended in 1 mL of diluent C, and 4 μL of PKH26 was mixed with 1 mL of diluent C. The two solutions were mixed together and incubated at ambient temperature for 5 min. Then 2 mL of FBS was added and incubated for 1 min, following which 4 mL of complete culture medium was added and centrifuged at 400 × g for 5 min. Cells were then transferred into another tube, washed three times, and finally resuspended in suitable medium. Polyethylene glycol (PEG) was used to induce cell fusion. In brief, both GES-1 cells (2 × 10^6^ cells) and CM-MSCs (1 × 10^7^ cells) were collected, washed with phosphate-buffered saline (PBS) and resuspended with 10 mL of PBS. The cells were mixed together, centrifuged at 400 × g for 5 min, and the supernatant was carefully removed. Cells were resuspended in 1 mL of PEG and incubated for 1 min. Then 20–30 mL of serum-free DMEM/F-12 culture medium was slowly added to the tube and the cells were carefully mixed. Cells were cultured at ambient temperature for 2 min, incubated at 37°C for 10 min, and centrifuged at 150 × g for 10 min. The supernatant was removed and the cells were resuspended in serum-free culture medium. CFSE^+^PKH-26^+^ cells were then sorted using FACS Aria (BD Biosciences, CA, USA). The sorted cells were resorted to ensure a purity of GSE^+^PKH-26^+^ cells >98%. Fused cells were then aliquoted into a 96-well plate after serial dilutions. Cells were examined at 12 h, and then every 24 h. Each well of the hybrids was deemed as a different clone, and each clone was then subcultured into a 24-well plate, a 6-well plate and then 25 cm^2^ flasks until they reached 90% confluency.

### Hematoxylin and eosin (H&E) staining and cytokeratin-18 (CK-18) immunofluorescence (IF) of GES-1 and hybrids

Slides with deposited GES-1 or hybrids were fixed with paraformaldehyde for 15 min at ambient temperature and washed three times with PBS for 5 min each. For H&E staining, standard protocols were applied after fixation of cells. For CK-18 IF slides, hydrogen peroxide (3%, v/v) was applied for 10 min after fixation of cells. After washing, the slides were blocked with 1% bovine serum albumin (BSA) solution for 20 min, and a mouse primary antibody against CK-18 (Santa Cruz Biotechnology CA, USA) was then applied, and slides were incubated overnight at 4°C and then washed with PBS. Fluorescein isothiocyanate (FITC)-conjugated anti-mouse IgG antibody of goat origin (Boster, China) was applied at room temperature and the slides were incubated in dark for 1 h. After washing, 20 μL of anti-fade mounting medium containing 4′,6-diamidino-2-phenylindole (DAPI) was applied. Slides were then cover-slipped and observed using a fluorescent microscope. Nuclear/cytoplasm ratio was analyzed using Image Pro Plus 6.0 (Media Cybernetics, MD) software.

### Flow cytometry analysis

CM-MSCs and hybrids were characterized by flow cytometry after staining with the following antibodies: CD45-FITC, CD34-FITC, CD105- Phycoerythrin (PE), CD73-PE, CD90-PE, HLA-ABC-FITC, HLA-DR- FITC (BioLegend, USA). In brief, 1 × 10^6^ cells were collected and washed twice with PBS, and resuspended in 50 μL of PBS. Antibodies were then added and incubated for 30 min, washed twice with PBS, and cells were resuspended with PBS and analyzed with FACS Calibur (BD Biosciences, USA).

### DNA ploidy analysis of hybrids

DNA ploidy analysis was performed to analyze the hybrids. Cells were harvested, washed twice with PBS, and fixed with 75% ethanol at 4°C overnight (>18 h). After centrifugation at 1000 rpm at 4°C, ethanol was removed, and cells were washed twice with cold PBS and incubated in 200 μl of RNase A (1 mg/mL) at 37°C for 30 min, following which 800 μl of propidium iodide (PI) was then added. The cells were mixed carefully, incubated in darkness for 30 min and analyzed with FACS Calibur flow cytometer (BD Biosciences, CA, USA). Turtle blood cells at G0/G1 phase were used as a control, and cell cycle phase distribution, DNA index, and percentage of cells in each phase were calculated using Modifit software (Verity Software House, ME, USA).

### Cell scratch test of hybrids

A scratch test was performed to assess the mobility of the hybrids. In brief, cells were incubated in a 6-well plate and at least five horizontal lines were drawn at the back of the plate. Then, 5 × 10^5^ cells were placed in each well and cultured overnight. Straight scratches were then made vertical to the line drawn on the back of the plate, and the wells were washed three times with PBS to remove excess cells. Cells were cultured with serum-free culture medium at 37°C with 5% CO_2_, and observed at 0 h, 24 h, 48 h, and 72 h.

### Transwell assay of hybrids

Transwell migration and invasion assays were performed to assess the migration and invasion of the hybrids, with six wells used per cell type. In brief, Matrigel was not applied in the migration assay, but in the invasive assay 3.9 μg/μL Matrigel was added to coat the microporous membrane (8 μm) in the upper compartment, and the chamber was incubated at 37°C for 30 min to effect gelation. Complete DMEM/F-12 culture medium with 10% FBS was added into the lower compartment. GES-1, CM-MSCs, and hybrids cells were harvested, washed and resuspended with serum-free culture medium. Then 1 × 10^5^ cells were added into the upper compartment and the cells were cultured at 37°C in 5% CO_2_. At the time of observation, the upper chamber was carefully removed from the well and its medium was removed. Residual cells were gently wiped from the upper chamber and the well was stained with hematoxylin. The number of cells that penetrated through the membrane was observed and counted in five fields, including the center of the membrane and four other random areas.

### Tetrazolium dye (MTT) colorimetric cell proliferative assay of hybrids

An MTT assay was performed to assess the proliferative activity of the hybrids. In brief, GES-1 cells, CM-MSCs, and the hybrids were harvested and aliquoted into a 96-well plate at 4,000 cells per well. The assay was performed at 24 h, 48 h, 72 h, and 96 h after culture. In brief, MTT (5 mg/mL) was added into each well (20 μL/ well) and cultured at 37°C for 4 h. The supernatant was carefully removed, and DMSO (200 μL/ well) was added. The plate was shaken for 15 min, and the absorbance (OD) was measured at 570 nm. The proliferation curve was generated using the equation: OD of hybrids/OD of control cells × 100%.

### Immunocytochemistry staining

Cells cultured on coverslip were fixed and stained for the expression of E-cadherin, N-cadherin and Vimentin. The slides were stained with mouse anti-human antibodies to E-cadherin (1:50), N-cadherin (1:50) and vimentin (1:100) (Santa Cruz Biotechnology, CA, USA). After washing, biotin-labeled goat anti-mouse IgG was added and streptavidin enzyme complex and DAB were applied (Boster, China). Cell positivity was multiplied by the intensity of staining and the percentage of stained cells to form a multiplicative score. The cases were sorted into four subgroups: H score 0–1 referred to negative expression; H score 2–3 to weak expression; H score 4–6 to moderate expression; and H score 6–9 to strong expression.

### Real-time quantitative PCR

Cells were homogenized and total RNA was isolated using an RNeasy Mini kit (QIAGEN China Co, China) following the manufacturer’s instructions. All samples were treated with RNase-free DNase (QIAGEN China Co, China). First-strand complementary DNA was made from total RNA using the Quantscript RT Kit (Tiangen Biotech, Beijing, China) according to the manufacturer’s instructions. To quantify the expression levels of Twist and Slug, real-time PCR amplifications were performed with the Opticon2 real-time PCR system (MJ Research, MA, USA). Real-time PCR assays were performed using SYBR Supermix-UDG (Tiangen Biotech, Beijing, China) in micro-reaction tubes. The PCR reaction was performed in a final volume of 20 μL, consisting of 10 μL of 2× SYBR Supermix, 1.0 μL of each 5′- and 3′- primer (10 pmol/μL), 3 μL of sample cDNA and 5.0 μL of ddH_2_O. All samples were run in triplicate. Glyceraldehyde-3-phosphate dehydrogenase (GAPDH) was used as an endogenous RNA reference gene. Primers and product sizes for Slug were upstream 5′-AACTACAGCGAACTGGACAC-3′, downstream 5′-AATGGAGCAGCGGTAGTC-3′, 143 bp. Primers and product sizes for Twist were upstream 5′-TTCTCGGTCTGGAGGATG-3′, downstream 5′-ACTGTCCATTTTCTCCTTCTC-3′, 129 bp. Primers and product sizes for GAPDH were upstream 5′-GAAGGTGAAGGTCGGAGTC-3′, downstream 5′-GAAGATGGTGATGGGATTTC-3′, 225 bp. The expression levels for each target gene were calculated using the comparative threshold cycle (CT) method. The ∆ct values were calculated according to the formula ∆ct = ct (gene of interest)-ct (GAPDH) in correlation analysis, and the 2-∆∆ct was calculated according to the formula ∆∆ct = ∆ct (control group)-∆ct (experimental group) for determination of relative expression. Data are represented as the mean ± standard deviation (SD) from three independent experiments.

### Tumor formation rate in nude mice

GES-1, CM-MSCs, and the hybrids were collected and 1 × 10^7^ cells were suspended in 100 μL of PBS. Cells were then injected subcutaneously into the armpit area of BALB/c nude mice (N = 8) and observed for 4 weeks for tumor formation. Samples of the subcutaneous mass were collected, and H&E staining and CK-18 IHC were performed for pathologic analysis of the mass.

### Statistical analysis

Statistical analysis was performed using SPSS 13.0 for Windows (SPSS Inc., IL, USA). For proliferation and transwell assays, values were expressed as means ± standard deviation (mean ± SD). Statistical significance between different cells groups was evaluated by ANOVA, followed by S-N-K’s post-hoc test. Fisher’s exact test was performed to test the frequency difference of immunocytochemistry staining (positive vs. negative) between different cells groups. A p-value < 0.05 was considered statistically significant.

## Results

### Hybrids acquired phenotype of both partner cells

**Figure 1:**
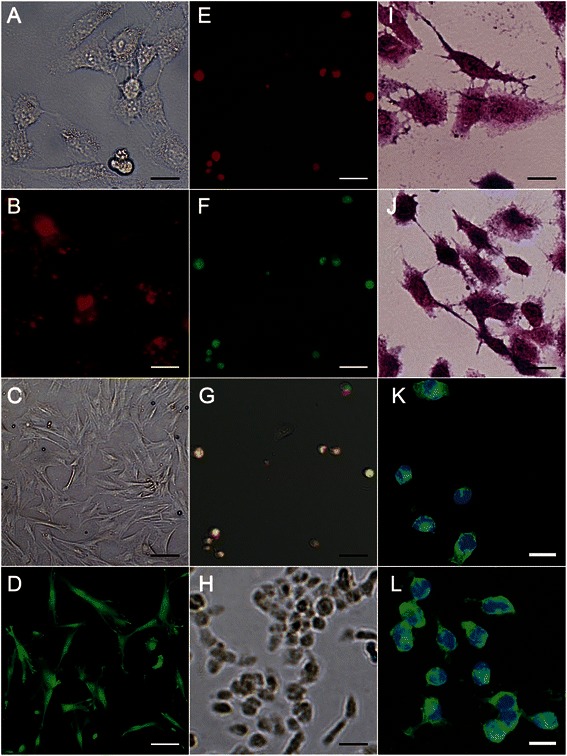
**GES-1 versus hybrids.** GES-1 **(A)** and CM-MSCs **(C)** were stained using PKH26 **(B)** and CFSE **(D)** separately. At day 1 after *in vivo* cell fusion and cell sorting, most cells expressed both PKH26 and CFSE **(E–G)**, and the hybrids began growing colonies at day 5 **(H)**. H&E staining showed that the morphologies of GES-1 **(I)** and fusion cells **(J)** were oval, spindle or polygonal, and CK-18 IF results showed that CK-18 was expressed in the cytoplasm in both GES-1 **(K)** and fusion cells **(L)**. Magnification: 400×, Scale bar **A**-**J** = 25 μm; **K**-**L** = 20 μm.

CM-MSCs were successfully cultured and collected. Expression of the most recognized surface antigen of CM-MSCs, including HLA-ABC, SH2 (CD105) and SH4 (CD73), was analyzed by flow cytometry, with 99.69%, 99.74%, and 99.73% positivity, respectively. Expressions of HLA-DR, CD45, and CD34 were also analyzed to assess hematopoietic stem cell contamination; expressions of these antigens were 0.01%, 1.59%, and 0.03% respectively. After GES-1 (Figure 1A, B) and CM-MSCs (Figure 1C, D) were labeled with PKH-26 and CFSE, *in vitro* cell fusion between GES-1 and CM-MSCs was performed.

CFSE^+^PKH-26^+^ cells were then sorted using FACS Aria (BD Biosciences, CA, USA). The fusion efficiency represented by double-positive cells was 5.77 ± 1.91%, as determined by fluorescence-activated cell sorting (FACS), and most of the cells expressed both PKH-26 and CFSE (Figure 1E–G) at day 1 after cell sorting. The hybrids began growing colonies at day 5. H&E staining showed that the morphologies of GES-1 cells (Figure 1I) and hybrids (Figure 1J) were oval, spindle-shaped and polygonal. Detection of CK-18 immunofluorescence indicated high-level expression of CK-18 in the cytoplasm of both GES-1 and the hybrids (Figure 1K–L). This observation indicates that the hybrids maintain the CK-18 characteristic of GES-1 cells. Both H&E and CK-18 IF results detected an increase in the nuclear/cytoplasm ratio in the hybrids (1.67 ± 0.24 for GES-1 vs. 0.83 ± 0.18 for GES-1, p < 0.05), which is a representative characteristic of tumor cells. CD90, which is characteristically expressed in CM-MSCs, was analyzed by FACS and found to be expressed at a low level (2.68%) in GES-1 cells, 28.76% in the hybrids, and at 19.36% in CM-MSCs. These results indicate that the hybrids acquired phenotypes from both parental cells. Compare to GES-1 cells, hybrids showed increased tumor-like characteristic.

### Hybrids showed ploidy disorder and increased metastatic and proliferation ability

**Figure 2:**
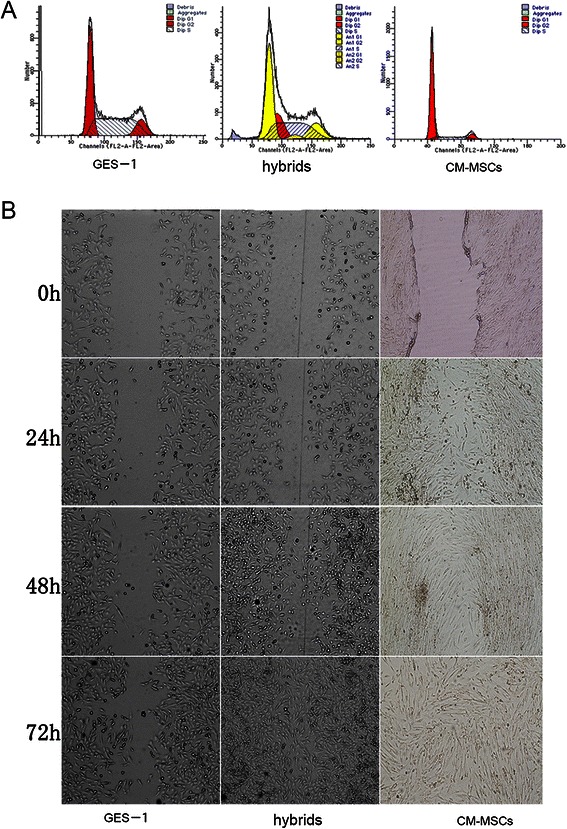
**DNA ploidy analysis and cell scratch assays. (A)** DNA ploidy analysis was performed on the parental and progeny cells. GES-1 and CM-MSCs were diploid. The majority of hybrids were aneuploidy cells (84.10%) (Figure 2A). The remainders were diploid (12.09%) and polyploid (3.81%). **(B)** Cell scratch results showed that hybrids had stronger migration ability than GES-1 cells. At 24 h, no significant difference was observed, but at 48 h, hybrids migrated toward the center of the scratch and almost filled the area. By 72 h, hybrids migrated toward the center and filled the area. CM-MSCs mirgrated fastest and filled the scratch at 48 h. Magnification: 100×, Scale bar 100 μm. Data are a representative of three experiments.

**Figure 3:**
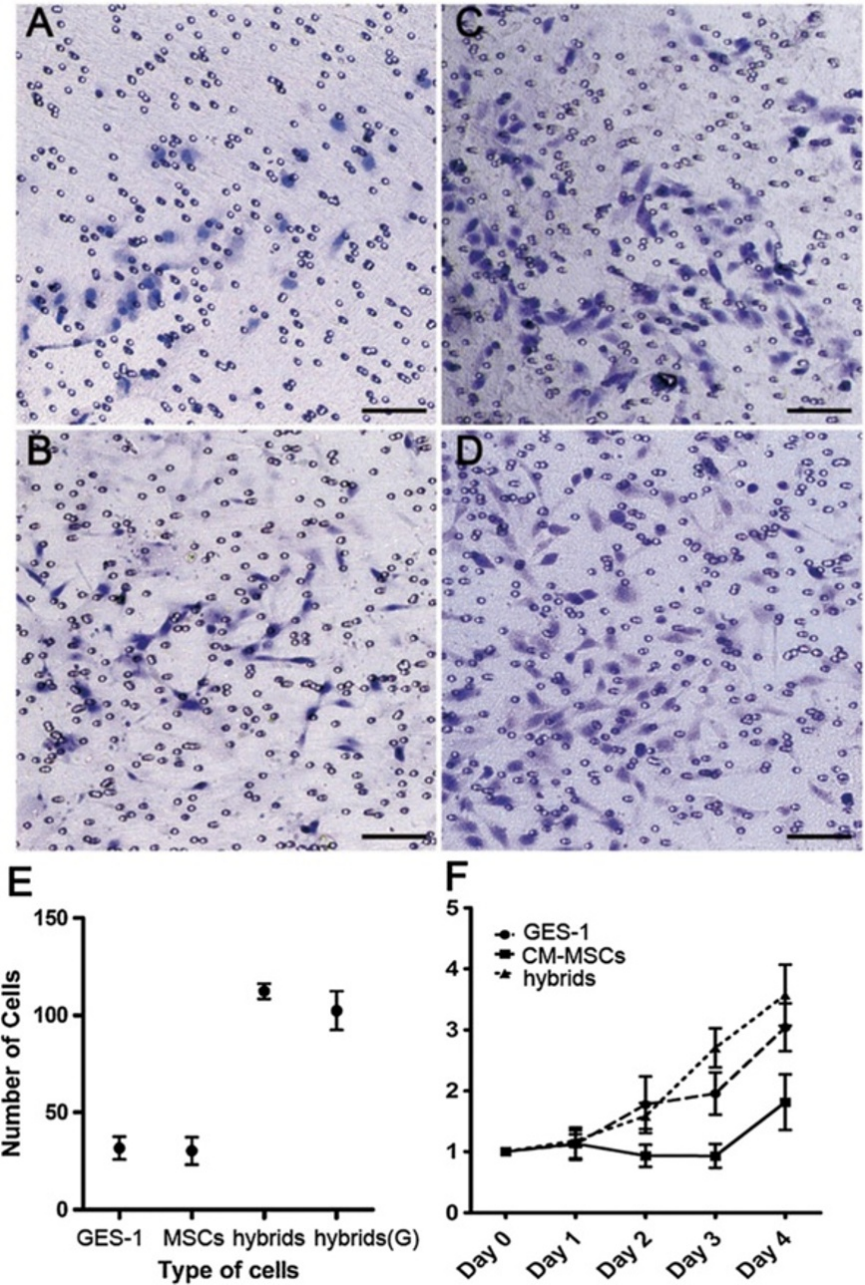
**Migration, invasion and proliferation of GES-1, CM-MSCs, and hybrids.** Transwell migration assay showed that GES-1 **(*panel A*)**, CM-MSCs **(*panel B*)**, and hybrids **(*panel C*)** could pass through the microporous membrane at 24 h, but only hybrids **(*panel D*)** could penetrate through Matrigel and cross the microporous membrane in the transwell invasion assay. Magnification: 100×, Scale bar 100 μm. Representative of three experiments are shown. **(*panel E*)** Graph indicates the number of cells crossing the microporous membrane in the transwell (hybrids vs. GES-1, p < 0.05; hybrids vs. CM-MSCs, p < 0.05), and number of hybrids penetrated Matrigel, indicated with hybrids(G). **(*panel F*)** Graph indicates the proliferation curve of GES-1, CM-MSCs, and hybrids (hybrids vs. GES-1, p > 0.05; hybrids vs. CM-MSCs, p < 0.05). Means ± SD of representative experiments are shown. A total of three experiments were performed.

DNA ploidy analysis was performed on the parental and progeny cells. GES-1 and CM-MSCs were diploid. The majority of hybrids were aneuploidy cells (84.10%) (Figure 2A). The remainders were diploid (12.09%) and polyploid (3.81%), a characteristic of tumor cells. In the cell scratch assay (Figure 2B) the hybrids had greater migration ability than GES-1. At 24 h, no significant difference was observed, but at 48 h the hybrids began to migrate toward the center of the scratch. By 72 h, the hybrids filled the scratch, while GES-1 cells migrated toward the center of the scratch but did not fill the area. CM-MSCs filled the scratch at 48 h. Furthermore, in the transwell migration assay, GES-1 (31.57 ± 15.55 cells/field) (Figure 3A), CM-MSCs (30.14 ± 18.75 cells/field) (Figure 3B), and hybrids (112.3 ± 10.36 cells/field) (Figure 3C) crossed the microporous membrane at 24 h, but in the transwell invasive assay only the hybrids cells (102.3 ± 24.33 cells/field) (Figure 3D) were able to penetrate the Matrigel coating and cross the microporous membrane. The numbers of migrated cells are significant difference as comparing hybrids to GES-1 and CM-MSCs (Figure 3E). These results indicate that fusion of GES-1 with CM-MSCs not only increase the migration ability, but also increase the invasive ability of the hybrids. MTT results show that the hybrids proliferate at a faster rate than GES-1 and CM-MSCs (Figure 3F). No significant difference between proliferation rates was observed on day 1 and 2, but the proliferation rate of the hybrids significantly increased at day 3 and day 4.

### Increased expression of EMT-related genes in hybrids

**Figure 4:**
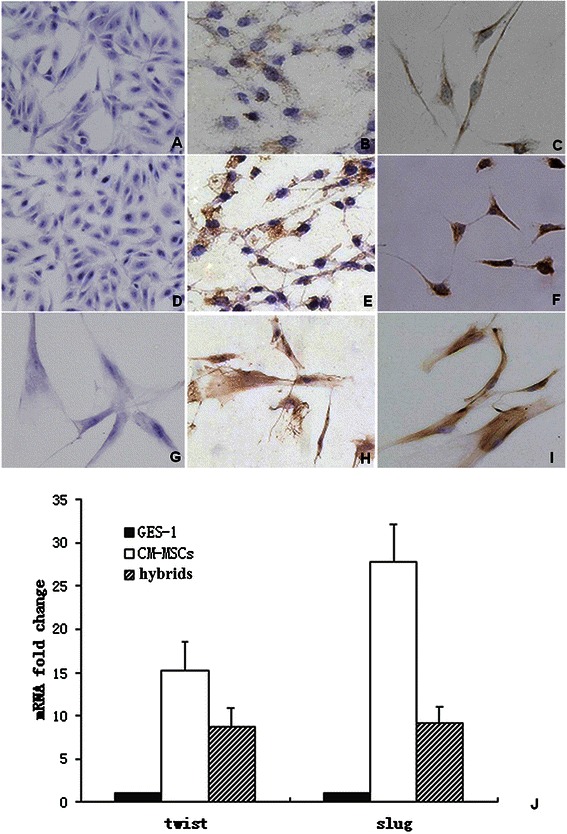
**Expression of EMT-related genes in hybrids.** Cells grown on coverslips were fixed and stained for the expression of E-cadherin, N-cadherin and vimentin. Magnification 200×. No E-cadherin expression was detected in all cells types **(*panel A***, GES-1; ***panel D***, CM-MSCs; ***panel G***, hybrids); N-cadherin expression was weak in GES-1 cells **(*panel B*)**, while CM-MSCs had strong expression **(*panel E*)**. The hybrids had moderate N-cadherin expression **(*panel H*)**. GES-1 cells expressed vimentin weakly **(*panel C*)**, while both CM-MSCs **(*panel F*)** and hybrids **(*panel I*)** had strong expression. Twist and Slug mRNA transcription were analyzed by quantitative RT-PCR. Compared with GES-1 cells, the mRNA expressions of Twist in CM-MSCs and fusion cells were upregulated by (15.2 ± 8.7)- and (8.7 ± 2.1)-fold, respectively; similarly, the mRNA expressions of Slug in CM-MSCs and fusion cells were upregulated by (27.8 ± 4.2)- and (9.2 ± 1.8)-fold, respectively (p < 0.05) **(*panel J*)**. Means ± SD of three representative results are shown.

EMT is characterized by the loss of epithelial marker E-cadherin and expression of mesenchymal markers including N-cadherin and vimentin [8]. Immunocytochemistry was performed to evaluate the expressions of E-cadherin, N-cadherin and vimentin here. GES-1, CM-MSCs and hybrids were all negative for E-cadherin (Figure 4A, D, G). GES-1 cells were weak for N-cadherin expression, CM-MSCs were strongly positive, and the hybrids had moderate expression (Figure 4B, E, H). For vimentin expression, GES-1 was weakly positive, and both CM-MSCs and the hybrids exhibited strong expression (Figure 4C, F, I). Real-time PCR was performed to measure the transcription of the EMT-related genes Twist and Slug in GES-1, CM-MSCs and fusion cells. Compared with GES-1, the mRNA expressions of Twist in CM-MSCs and fusion cells were upregulated by (15.2 ± 8.7)- and (8.7 ± 2.1)-fold, respectively. Similarly, the mRNA expressions of Slug in CM-MSCs and fusion cells were upregulated by (27.8 ± 4.2)- and (9.2 ± 1.8)-fold, respectively (p < 0.05) (Figure 4J).

### Increased proliferation and tumorigenicity in hybrids

**Figure 5:**
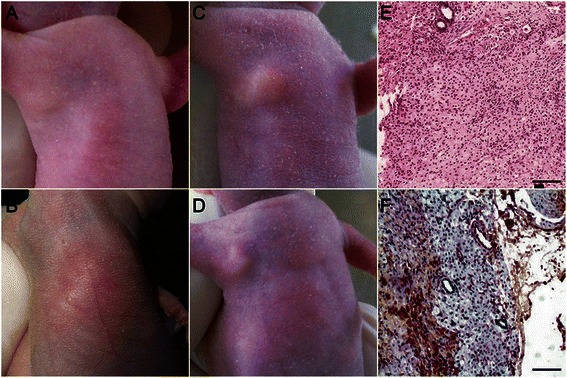
**Subcutaneous injection of the hybrids.** At 7 days after subcutaneous injection in BALB/c nude mice, no mass was observed in those injected with GES-1 **(*panel A*)** or CM-MSCs **(*panel B*)**, but masses were observed in mice injected with hybrids **(*panel C*)**. With prolonged observation time, GES-1 and CM-MSCs remained negative for subcutaneous masses, while the size of the masses decreased in mice injected with hybrids **(*panel D*)**. H&E staining **(*panel E*)** and CK-18 IHC **(*panel F*)** results show that the masses were of epithelial origin and gastric gland structures were observed, but not show characteristics of malignant tumor. Magnification: 40×, Scale bar 200 μm. Data are representative of three experiments.

GES-1, CM-MSCs, and the hybrids were collected and 1 × 10^7^ cells were injected subcutaneously into the armpit area of BALB/c nude mice (N = 8) and mice were observed for 4 weeks for tumor formation. At 7 days after injection, no mass was observed in those injected with GES-1 (Figure 5A) or CM-MSCs (Figure 5B), However, subcutaneous masses were observed in six of the eight mice injected with hybrids cells 7 days after injection (Figure 5C). With prolonged observation time, GES-1 and CM-MSCs group remained negative for subcutaneous masses , and the volume of four of the six masses observed for hybrids group decreased (Figure 5D). H&E staining (E) and CK-18 IHC (F) results showed that the masses were of epithelial origin and gastric gland structures were observed, but no characteristics of malignant tumor was found. This result indicated that the tumorigenicity potential of normal epithelial cells increased after fusion with MSCs, which suggests that cell fusion could participate in gastric cancer carcinogenesis.

## Discussion

Mutations are believed to be the principal mechanism of malignant transformation [9]. However, the discovery of cancer stem cells and tumor heterogeneity suggests that as the main characteristic of malignant tumors, the ability of invasion and metastasis is not determined solely by genetic alterations. Recent evidence indicates that alternative mechanisms, such as cell-cell fusion, may also render cells with the ability to escape cell cycle control, tissue invasion, and metastasis [10]. Herein, we report that fusion between immortalized non-tumorigenic human gastric epithelial cells with CM-MSCs can produce transformed hybrids and induce epithelial-to-mesenchymal transition.

Cell fusion of normal mammalian somatic cells is a tightly controlled process that is restricted to a few cell types, and results in terminally differentiated multinuclear cells. However, cell fusion causes chromosomal aberrations, which can have potential pathological consequences. Cancer stem cells have been discovered in solid tumors and are believed to initiate and sustain neoplastic growth; however, the origin of these cells remains to be debated [11,12]. Normal stem cells with inherent self-renewal capacity may acquire mutations and become transformed. The rareness of tissue stem cells may counter this theory because of the low probability that they could be targeted by mutations. Differentiated tissue cells, especially epithelial cells in gut, undergo rapid turnover and rarely accumulate enough mutations to become transformed. He *et al*. proposed a stem cell fusion model where abnormal or pre-malignant somatic cells, including benign tumor cells, fuse with BMDSCs (including MSCs) to form malignant hybrids that can promote carcinogenesis [2]. Conceptually, cell fusion between stem cells and mutated differentiated cells might lead to the acquisition of self-renewal capacity that allows further accumulation of transforming mutations. In the present study, we used hybrids established from *in vitro* cell fusion between GES-1 and CM-MSCs to investigate whether cell fusion results in carcinogenesis. Hybrids acquired both CK-18 and CD90 phenotypes from both parent cells, GES-1 and CB-MSC, and also carried the characteristics of tumor cells, such as an increased nuclear/cytoplasm ratio, increased proliferation rate and aneuploidy. Migration and invasive ability of the hybrids significantly increased *in vitro* and the hybrids were able to form subcutaneous mass of epithelial origin *in vivo*. These results indicate that BMDSCs acquire an epithelial phenotype through cell fusion, and that cell fusion may be the mechanism for gastric epithelial cells to acquire metastatic ability. In contrast to the observation of Wang *et al.*, which showed that fusion between esophageal carcinoma cells and CM-MSCs suppressed tumorigenicity [13], our results indicate that tumorigenicity of the hybrids was superior to GES-1 cells, indicating that cell fusion may participate in gastric carcinogenesis. These contradictions could be due to differences in genetic background between the fusion partners. In our study, the fusion partner for CM-MSCs was immortalized non-tumorigenic GES-1 cells, whereas malignant tumor cells (EC9706 or KYSE150) were used by Wang *et. al.* Further studies are needed to determine the role of fusion between BMDCs and tissue cells in different disease conditions.

EMT is an evolutionarily conserved process that occurs during development and may also be involved in cancer. EMT generates cells with properties of stem cells and contributes to tumor progression and metastasis [14,15]. Previous studies have shown that breast cancer stem cells display EMT characteristics and EMT plays a major role in sustaining CSCs [16]. Several genes encoding transcription factors, such as Twist, Snail and Slug, have been shown to govern EMT in normal and transformed epithelial cells [17]. During EMT, epithelial cells lose apicobasal polarity and intercellular junctions penetrate into the extracellular matrix-rich compartment. E-cadherin is a key component of adherens junctions and the suppression of E-cadherin and a switch to the expression of mesenchymal cadherins, such as N-cadherin, are characteristics of EMT that are associated with tumor invasion [18]. In cancer, EMT is thought to be induced by signals from the stroma associated with tumors, such as hepatocyte growth factor, platelet-derived growth factor, and transforming growth factor-beta [19,20]. Herein, we show that fusion between epithelial cells with MSCs cells may directly result in EMT of the hybrids. Powell *et al.* reported that fusion between intestinal epithelial cells with macrophages induce EMT, which increased the migration and invasion ability both *in vitro* and *in vivo* [21]. Therefore, cell fusion between MSCs with mutated epithelial cells could link the origin of cancer stem cells with EMT, and require further investigation. Furthermore, during tumor progression, cell fusion between neoplastic cells with stroma MSCs may be one of the driving forces of clone evolution and contribute to tumor heterogeneity.

## Conclusions

The present study showed that fusion of gastric epithelial cells with mesenchymal stem cells resulted in EMT and malignant transformation. However, the proposal that cell fusion can initiate malignant transformation by no means excludes other mechanisms. We argue that cell fusion between MSCs and epithelial cells may be one of the mechanisms of EMT and malignant transformation.
